# The Interplay between Protein L-Isoaspartyl Methyltransferase Activity and Insulin-Like Signaling to Extend Lifespan in *Caenorhabditis elegans*

**DOI:** 10.1371/journal.pone.0020850

**Published:** 2011-06-13

**Authors:** Shilpi Khare, Carole L. Linster, Steven G. Clarke

**Affiliations:** 1 Department of Chemistry and Biochemistry, University of California-Los Angeles, Los Angeles, California, United States of America; 2 Molecular Biology Institute, University of California-Los Angeles, Los Angeles, California, United States of America; 3 Laboratory of Physiological Chemistry, de Duve Institute and Université catholique de Louvain, Brussels, Belgium; University of Washington, United States of America

## Abstract

The protein L-isoaspartyl-O-methyltransferase functions to initiate the repair of isomerized aspartyl and asparaginyl residues that spontaneously accumulate with age in a variety of organisms. Caenorhabditis elegans nematodes lacking the pcm-1 gene encoding this enzyme display a normal lifespan and phenotype under standard laboratory growth conditions. However, significant defects in development, egg laying, dauer survival, and autophagy have been observed in pcm-1 mutant nematodes when deprived of food and when exposed to oxidative stress. Interestingly, overexpression of this repair enzyme in both Drosophila and C. elegans extends adult lifespan under thermal stress. In this work, we show the involvement of the insulin/insulin-like growth factor-1 signaling (IIS) pathway in PCM-1-dependent lifespan extension in C. elegans. We demonstrate that reducing the levels of the DAF-16 downstream transcriptional effector of the IIS pathway by RNA interference reduces the lifespan extension resulting from PCM-1 overexpression. Using quantitative real-time PCR analysis, we show the up-regulation of DAF-16-dependent stress response genes in the PCM-1 overexpressor animals compared to wild-type and pcm-1 mutant nematodes under mild thermal stress conditions. Additionally, similar to other long-lived C. elegans mutants in the IIS pathway, including daf-2 and age-1 mutants, PCM-1 overexpressor adult animals display increased resistance to severe thermal stress, whereas pcm-1 mutant animals survive less long under these conditions. Although we observe a higher accumulation of damaged proteins in pcm-1 mutant nematodes, the basal level of isoaspartyl residues detected in wild-type animals was not reduced by PCM-1 overexpression. Our results support a signaling role for the protein L-isoaspartyl methyltransferase in lifespan extension that involves the IIS pathway, but that may be independent of its function in overall protein repair.

## Introduction

A master regulator of aging in the nematode *Caenorhabditis elegans* is the insulin/insulin-like growth factor-1 signaling (IIS) pathway. The IIS pathway is conserved in other organisms, including *Drosophila melanogaster* and mammals [1]. Activation of the DAF-2 insulin-like receptor in *C. elegans* triggers the successive phosphorylation and activation of several kinases including AGE-1 (Class 1A phosphatidylinositol-3-kinase) and AKT (protein kinase B); the activated AKT kinase can subsequently phosphorylate the FoxO transcription factor DAF-16, excluding it from the nucleus and thereby rendering it inactive [2], [3]. However, when this kinase cascade of phosphorylation events is deactivated, the DAF-16 transcription factor enters the nucleus, leading to the enhanced expression of genes involved in longevity, dauer formation, and stress resistance [2], [3]. *C. elegans* mutants lacking a functional insulin-like receptor or PI3-kinase (*daf-2* and *age-1* mutants, respectively), display lifespan extensions spanning two-fold to ten-fold and show enhanced stress resistance [4]–[6]. The long-lived phenotype for the *daf-2* pathway mutants is abolished upon additional mutation of the DAF-16 transcription factor [5], [7], [8].

Another protein implicated in *C. elegans* longevity is the protein L-isoaspartyl (D-aspartyl)-*O-*methyltransferase (PCM-1), the product of the *pcm-1* gene [9]. Known primarily for its protein repair role in higher organisms, this highly conserved enzyme specifically recognizes damaged aspartyl and asparaginyl residues that accumulate with age [10]. Following the spontaneous deamidation and/or isomerization of these residues, protein backbones become kinked. The repair enzyme methylates the α-carboxyl group of isoaspartyl residues, thereby facilitating “unkinking” of the backbone and conversion of the isomerized residues back to normal L-aspartyl residues [10]. *E. coli* lacking the isoaspartyl methyltransferase gene display increased susceptibility to various environmental stresses [11]. Knockout mice in the corresponding *Pcmt1* gene accumulate altered proteins, show growth retardation, and are subject to premature death from fatal seizures at an average age of 42 days [12]. In plants, absence of the repair methyltransferase has been shown to be involved in decreased seed longevity and germination vigor in *Arabidopsis thaliana* [13]. On the contrary, overexpression of the methyltransferase in *E. coli* results in increased survival under heat shock [14] and oxidative stress [15] and extended lifespan under thermal stress in *D. melanogaster* [16] and *C. elegans* [17].

Further characterization of this repair enzyme has revealed interplay between PCMT and major intracellular signaling pathways, including the IIS pathway and the mitogen-activated protein kinase (MAPK) pathway. For example, *Pcmt1* knockout mice showed activation of the IIS signaling pathway as evidenced by increased phosphorylation of kinases including AKT, glycogen synthase kinase-3 beta (GSK-3β), and PDK-1 [18]. In relation to the MAPK pathway, PCMT1-knockdown human cells stimulated with EGF (epidermal growth factor) showed increased phosphorylation of MAPK pathway components, including Raf-1, MEK, and ERK1/2 [19], [20]. Additionally, Doyle and colleagues found that the absence of PCMT1 in mammalian lymph node cells causes T-cell hyperproliferation and increased phosphorylation of MAPK pathway components MEK1/2, ERK1/2, and RSK1 in response to CD3 receptor stimulation [21]. In contrast, enhanced PCMT1 expression induced by both valproic acid and lithium was accompanied by inactivation of GSK-3β in astrocytoma and neuroblastoma cells [22], [23]. These findings illustrate that, in addition to protein repair, PCMT1 might be directly or indirectly involved in the regulation of the IIS pathway and/or the MAPK pathway in mammalian systems.

As opposed to mice, effects of a deficiency in the protein L-isoaspartyl-*O*-methyltransferase resulting from mutations in the *pcm-1* gene in *C. elegans* become apparent only under certain stress conditions. In control conditions, *pcm-1* mutants display a similar lifespan and brood size when compared to wild-type (N2) animals [24]. Under starvation conditions, *pcm-1* mutants display defects in dauer formation and survival as well as L1 larvae survival [17], [25]. During dauer formation, *pcm-1* mutants display reduced levels of autophagy, indicative of enhanced insulin/TOR signaling in the absence of PCM-1 [25]. Furthermore, under oxidative stress conditions, we have recently shown that *pcm-1* mutants display defects in egg laying as well as delayed larval development [26]. An additional mutation in the *daf-2* gene in the *pcm-1* mutants restored a normal dauer lifespan [17] and recovered stress defects in larval development and egg laying [26], indicating an epistatic relationship between genes of the IIS pathway and the gene encoding the isoaspartyl methyltransferase.

Given the normal adult lifespan of *pcm-1* mutant *C. elegans*, it was a surprise to find that PCM-1 overexpression extends adult lifespan two-fold compared to wild-type nematodes under mild thermal stress [17]. As it was previously shown that the lifespan extension of long-lived IIS pathway mutants is DAF-16-dependent, this work is focused on determining whether the prolonged lifespan of PCM-1 overexpressor animals also involves the transcriptional activity of DAF-16. Furthermore, the importance of the protein repair function of PCM-1 in this context has been addressed.

## Methods

### Worm strains and procedures

Standard procedures used to maintain *C. elegans* strains were adapted from Sulston and Hodgkin [27]. The N2 strain was obtained from the *Caenorhabditis* Genetics Center (St. Paul, MN). Two transgenic strains (*PL51* and *PL54)* along with the *pcm-1 (qa201)* mutant strain were used for all experimental procedures [17]. Strain *PL51* is a non-integrated strain that was created by injection of a plasmid expressing the *pcm-1* gene under its endogenous promoter into a *pcm-1* mutant background. Three to seven-fold higher isoaspartyl methyltransferase activities were measured in transgenic *PL51* worm extracts compared to wild-type N2 extracts prepared from animals grown in liquid culture [26]. Strain *PL54* contains a similar plasmid (also in a *pcm-1* mutant background) to the *PL51* strain except that it encodes a PCM-1 protein with two mutated residues in the enzyme's AdoMet cofactor binding site (*G88V* and *G90V)*. Extracts prepared from *PL54* nematodes did not demonstrate any methyltransferase activity [26]. The *pcm-1* (*qa201*) mutant lacks exons 2–5 of the *pcm-1* gene [24] and alternative exon 1 from the partially overlapping *C10F3.4/mcp-1* gene [26]. This allele was backcrossed eight times into an N2 background [17] to obtain the *pcm-1* (*qa201*) strain, which also totally lacks methyltransferase activity. Protein L-isoaspartyl *O*-methyltransferase activity was measured again in each of the strains used in this study and values are provided in Table 1. The integrated DAF-16::GFP roller strain *TJ356* was used to test *daf-16* RNAi efficacy and to monitor subcellular DAF-16 localization under various conditions. This strain was kindly given to us by Dr. Brian Head (UCLA Department of Biological Chemistry). In the absence of *daf-16* RNAi, we confirmed expression of the GFP-tagged DAF-16 protein in most cell types (including the hypodermis, neurons, intestine, and gonad) except the pharynx in the *TJ356* strain [28]. With *daf-16* RNAi, we observed a loss of fluorescence in all the *daf-16* expressing regions except for neurons. The efficiency of *pcm-1* RNAi was tested with the *xtEx104 (UZ122)* strain expressing a PCM-1::GFP fusion protein [25]. In the absence of *pcm-1* RNAi, we confirmed PCM-1 expression (body wall, reproductive tissues, and neurons) as initially reported by Gomez *et al*. [25] and observed a decrease in fluorescence (except in neurons) following *pcm-1* RNAi treatment in the *UZ122* strain. All strains were kept at 20°C or 25°C on nematode growth medium plates streaked with *E. coli* OP50 (NGM + OP50) [27].

**Table 1:** PCM-1 activity measurements in various *C. elegans* strains.

Strain	Methyl Groups Transferred (pmol/min/mg protein)
N2	0.60±0.04
*pcm-1(qa201)*	0.01±0.03
*PL51*	1.97±0.26
*PL54*	−0.10±0.01

Nematode protein extracts were prepared from liquid culture and analyzed for PCM-1 methyltransferase activity as described by Kagan *et al.* (1997). Protein extracts were incubated with the methyl-accepting peptide substrate KASA-isoD-LAKY (100 µM) and 20 µM [^14^C]-AdoMet at 30°C for 2 h. Control incubations were conducted as above except that KASA-isoD-LAKY was not added to the reaction. Protein L-Isoaspartyl *O-*methyltransferase activity levels are given as the amount of methyl groups transferred after correction for the background levels measured in the absence of KASA-isoD-LAKY for the various *C. elegans* strains used in the lifespan analyses, thermotolerance assays, RT-qPCR analyses, and isoaspartyl quantifications performed in this study. The results shown are means (± SD) of two separate activity measurements conducted with one set of lysates.

### Lifespan analysis and RNAi

The protocol used to complete the lifespan studies was adapted from Sutphin and Kaeberlein [29]. First, eggs from three strains (N2, *PL51*, and *PL54*) were collected following hypochlorite treatment of gravid adults and were transferred to NGM + OP50 plates. Following an approximately 48 h incubation at 20°C, L4 larvae from each strain were transferred to NGM plates containing isopropyl β-D-1-thiogalactopyranoside at 1 mM (*pcm-1* RNAi) or 2 mM (*daf-16* RNAi) (IPTG; Anatrace, Inc. #I-1003), 0.025 mg/ml carbenicillin sodium salt (CB; Sigma #C-1389), and 45 µM 5-fluorodeoxyuridine (FUDR; Sigma #46875) and streaked with RNAi-expressing bacteria (NGM + IPTG + CB + FUDR + RNAi). Following transfer (day 0), lifespan plates were incubated at 25°C until final scoring for survival was completed (survival was scored every other day until all animals were dead). Animals were considered dead when no signs of viability (motility, pharyngeal pumping, and response to platinum wire prodding) were detec. Lifespan experiments were repeated three times. Averages of mean and maximum survival were determined and examined for statistical significance using the ANOVA test for significance.

The RNAi bacteria (either scrambled control or *daf-16* (I-5m24)) were obtained from the Ahringer library [30] kindly provided by Dr. Brian Head at the UCLA Department of Biological Chemistry. The *pcm-1* RNAi feeding clone, derived from the *C. elegans* ORFeome Library v1.1 [31], was obtained from Open Biosystems (Huntsville, AL). Several clones were derived from the original bacterial stock; the clones #2 and #3 used in this study are lacking exon 3 or part of exon 2 of the *pcm-1* (*C10F3.5a*) open reading frame, respectively. The identity of the RNAi clones was verified by sequencing using the sjj_R13H8.1 primers (listed on www.wormbase.org as sjj_R13H8.1_f: AGTACAGCAATTCCCAAATGAAA and sjj_R13H8.1_b: ATTGGATTTCGAAGfAAGTGGAT) for the *daf-16* gene and the pL4440-dest-RNAi universal primers (For: GTTTTCCCAGTCACGACGTT, Rev: TGGATAACCGTATTACCGCC) for the *pcm-1* gene. For lifespan experiments in *C. elegans*, individual RNAi bacterial colonies were grown in LB + 50 µg/mL ampicillin for 7 h at 37°C; bacteria were then seeded onto NGM + IPTG + CB + FUDR plates and allowed to grow for 72 h before L4 larvae transfer.

### Thermotolerance assays

The protocol developed for this assay was adapted from Lithgow *et al*. [32], except that day 1 adult animals were tested at 37°C instead of 35°C. L4 larvae from each strain were transferred (20–30 animals per trial) to NGM + OP50 plates and were placed at 20°C overnight. The next morning, plates were transferred to 37°C and, at the inffdicated times, nematodes were scored for viability (motility, pharyngeal pumping, and response to platinum wire prodding). Animals were considered dead when no signs of viability were detectable. The assay was repeated in triplicate and significance of survival time points was determined using the ANOVA test for significance.

### Microscopy Assays

To monitor the subcellular localization of DAF-16 under various experimental conditions, the *TJ356* strain was used. For the microscopy assays at 25°C, the progeny of RNAi-fed *TJ356* L4 larvae were grown at 25°C until adulthood in both control and *pcm-1* RNAi conditions and were scored for DAF-16 localization as done previously by Henderson and Johnson [28]. For the microscopy assays at 37°C, the progeny of RNAi-fed *TJ356* L4 larvae were grown in the same manner, except that animals were kept at 20°C. At day 1 of adulthood, the control RNAi-treated and *pcm-1* RNAi-treated nematodes were incubated at 37°C for two hours and DAF-16 subcellular localization was determined [28].

### Reverse transcription and quantitative real-time PCR

#### RNA extraction and reverse transcription

For all four stains tested (N2, *PL51*, *PL54*, *pcm-1 (qa201)*), gravid adults were treated for 3 min in a final concentration of 1.9 % sodium hypochlorite and 0.3 M NaOH. After five washes with M9 media, eggs were collected and starved overnight in M9 media to obtain synchronized L1 larvae. The L1 larvae were then transferred to NGM + OP50 plates and were incubated at 25°C for 48 h until nematodes had reached day 1 of adulthood. Day 1 adults were collected in M9 media (n = 200, triplicate samples for each strain) and stored at −80°C. For total RNA extraction, animals were homogenized in 1 mL TRI Reagent® (Molecular Research Center, #TR-118) with 1% β-mercaptoethanol (BioUltra, Sigma, #63689) using a Polytron PT2000 homogenizer with a 7 mm generator. Phase separation of RNA from DNA and proteins was completed using bromochloropropane (Molecular Research Center, #BP-151). RNA was DNAse-treated and further purified using the RNeasy Fibrous Tissue Mini Kit (Qiagen; Cat #74704). Following quantitation of RNA by measuring the absorbance at 260 nm using a NanoDrop spectrophotometer (the yield was generally about 4 µg RNA per 200 animals) and verification of absence of protein contamination by 260/280 nm ratio determination (between 1.8 and 2.0), a sample was run on a 1% TAE agarose gel to check for RNA degradation. RNA integrity was noted by observation of two sharp and discrete 26S and 18S ribosomal bands and the absence of leading smears, genomic DNA, and extra bands in each well. DNA-free RNA (2 µg in a total volume of 40 µl) was then converted to complementary DNA using oligo (dT) primers and a two-step RT-PCR procedure with heat denaturation (RETROscript Kit, Ambion #AM1710) according to the manufacturer's instructions.

#### Quantitative real-time PCR and qPCR primers

Amplification reactions were carried out in a total volume of 20 µl containing the SYBR Premix Ex Taq reagent (Clontech Laboratories, Inc., Takara Product #TAK RR041A), 0.3 µM of gene-specific forward and reverse primers, and cDNA corresponding to 100 ng input RNA in the reverse transcriptase reaction. The reaction conditions for the Opticon 2 system (MJ Research) were: 95°C for 5 min, followed by 40 cycles of 95°C for 10 s, 60°C for 20 s, 72°C for 20 s. The fluorescence was measured at each cycle at 80°C. Melting curves were performed after the PCR to assess the presence of a unique final product, and for each primer pair the product from one reaction was run on an agarose gel to further verify specificity and to confirm the expected band size.

The gene expression data are presented as the -fold change in mRNA transcript abundance in transgenic or mutant worm strains, normalized to two endogenous reference genes (*cdc-42* and *ama-1*), relative to the wild-type N2 strain. *Cdc-42* encodes the RHO GTPase and *ama-1* encodes RNA polymerase II; both genes were previously validated as relatively reliable reference genes to use for quantitative gene expression studies in *C. elegans* [33]. The expression results were similar when normalized against *cdc-42* or *ama-1* individually. Normalized -fold changes (and statistical significance) were calculated using the REST 2009 software (Qiagen).

Quantitative real-time PCR primers were newly designed for the *C. elegans* genes *ama-1* (forward primer: 5′-CGGTCAGAAAGGCTATCGAG-3′; reverse primer: 5′-CCAACCTCCTGACGATTGAT-3′), *pcm-1* (forward primer: 5′-TGTATGGCAATGATGGTTGG-3′; reverse primer: 5′-ACGACCGTCTCCCTCAATAA-3′), and *hsp-12.6* (forward primer: 5′-GTGATGGCTGACGAAGGAAC-3′; reverse primer: 5′-GGGAGGAAGTTATGGGCTTC-3′). Previously published primer pairs were used for the *C. elegans* genes *cdc-42* [33], *sod-3*, *daf-16* [34], *mtl-1*, *dod-3*, *F21F3.3*, *dod-22*, and *dod-24* [35].

### Quantification of protein substrates for the L-isoaspartyl methyltransferase in *C. elegans* extracts

Nematodes from each strain (N2, *PL51*, and *pcm-1 (qa201)*) were grown at either 20°C or 25°C for 5 days in liquid cultures (S-media supplemented with concentrated *E. coli* OP50) and harvested by washes and sucrose flotation as previously described [24]. Preparation of *C. elegans* cytosol and determination of protein concentration therein was also done as previously [24]. To quantify the level of isoaspartyl methyltransferase substrates in the various protein extracts, base-labile methyl ester groups were assayed in those extracts by a method modified from that of Kagan *et al*. [24]. Specifically, nematode cytosolic extracts (125 µg protein) were incubated in a 50 µl reaction mixture of the following composition: 0.1 M Bis-Tris (pH 6.4) buffer, 0.67 µM *S-*adenosyl-*L*-[*methyl*-^3^H]methionine (Perkin Elmer, 78 Ci/mmol), and 2.9 µg human recombinant L-isoaspartyl methyltransferase (this enzyme preparation had a specific activity of 19.5 nmol min^−1^ mg^−1^ when the peptide KASA-isoD-LAKY was used as a substrate). The reaction mixtures were incubated for 2 h at 30°C and were quenched via addition of 50 µl 2X Laemmli sample buffer containing 10% β-mercaptoethanol followed by heating at 70°C for 8 min. Approximately 40 µg of the polypeptides within the above reaction mixtures were then separated by gel electrophoresis using NuPAGE® Novex 4–12% SDS-PAGE gels (pH = 7) (Catalog # NP0335) and NuPAGE® MES SDS Running Buffer (Catalog #NP002) from Invitrogen. The level of base-labile [^3^H]-methyl esters was assayed in 3 mm gel slices by a vapor diffusion method as described by Kagan *et al.* [24], except that 150 µl of 1 N sodium hydroxide were added to the gel slices and that scintillation vials were incubated at 38°C for 24 hours before counting. Methyl-accepting proteins were quantified in two or three biological replicate extracts for each worm strain tested.

## Results

### PCM-1 overexpression extends lifespan in a DAF-16-dependent manner

It has previously been shown that PCM-1 overexpression extends the lifespan of adult nematodes under mild thermal stress [17]. Nematodes expressing a PCM-1 protein with a mutated AdoMet-binding site in a *pcm-1* null background (*PL54* strain) had a lifespan that was similar to wild-type (N2) animals under the same conditions indicating that the lifespan extension of the overexpressor (*PL51*) strain was dependent on the methyltransferase activity of PCM-1 [17]. We confirm here that the lifespan extension observed in the *PL51* strain is dependent on PCM-1 overexpression, as the lifespan of *PL51* adult nematodes fed with two different clones of *pcm-1* RNAi was comparable to that of wild-type nematodes treated with control RNAi (Figure S1). The lifespan of wild-type nematodes was not significantly affected by *pcm-1* RNAi.

**Figure 1:**
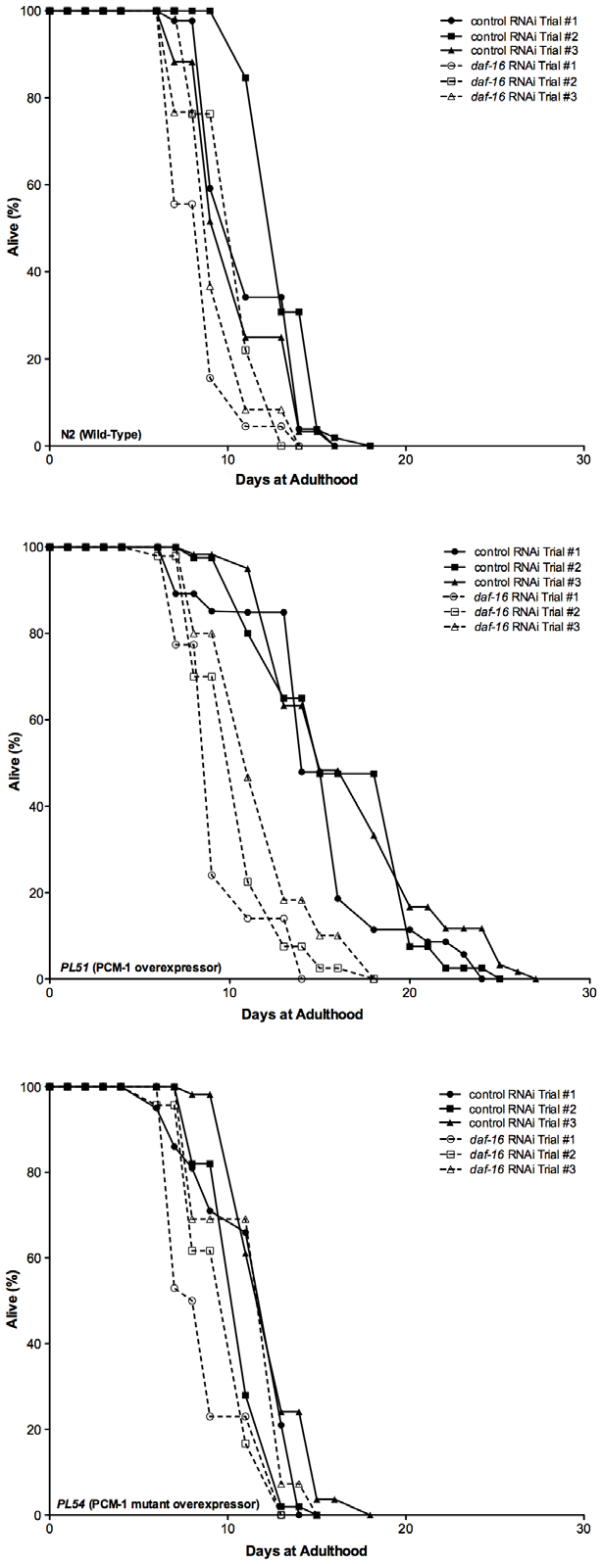
PCM-1 overexpression extends lifespan in a *daf-16*-dependent manner under mild thermal stress (25°C). Lifespan analyses (three trials) were completed at 25°C for three nematode strains (N2 wild-type (top panel), wild-type PCM-1 overexpressor strain (*PL51*) (middle panel), mutant PCM-1 overexpressor strain (*PL54*) (bottom panel)) grown in two conditions: control RNAi (solid lines) and *daf-16* RNAi (dotted lines). L4 larvae were transferred (Day 0) to NGM plates streaked with RNAi bacteria and survival was scored every other day until all nematodes were dead. Statistical analysis of these data is given in Table 2.

To test whether the PCM-1 overexpression effect was due to a modulation of the IIS system, we tested the dependence of this effect on the presence of DAF-16, the critical downstream transcription factor of the IIS. Specifically, we grew the N2, *PL51*, and *PL54* strains on bacteria expressing scrambled control RNAi or *daf-16* RNAi and used FUDR to prevent egg laying and cross generation sampling [29], [36]. We confirmed that PCM-1 overexpressing animals display on average an increase of 38% and 51% in median and maximum lifespan, respectively, compared to wild-type animals, and that no change in survival is observed between wild-type animals and nematodes expressing a catalytically inactive PCM-1 enzyme (Fig. 1, Table 2). In all strains tested, the lifespan values obtained in this study in the presence of FUDR were slightly reduced compared to those obtained previously in the absence of FUDR by Banfield *et al*. [17], but the same lifespan patterns were observed on control RNAi bacteria (Fig. 1, Table 2). All lifespan extensions (including the 50%, 25% and 10% survival values) measured for the overexpressor strain were statistically significant when compared to wild-type (N2) animals (Table 2).

**Table 2:** PCM-1 overexpression extends *C. elegans* lifespan in a DAF-16-dependent manner under mild thermal stress (25°C).

Strain	n	50% Survival (Hours)	25% Survival (Hours)	10% Survival (Hours)	Maximum Lifespan (Hours)
N2	152	2.3±0.9	3.2±0.8	4.2±1.1	5.0±0.8
*pcm-1 (qa201)*	136	1.7±0.6(*p = *0.04)**	2.8±0.6 (*p* = 0.003)**	3.3±1.0 (*p* = 0.01)**	4.0±1.0
*PL51*	142	3.7±0.6	4.5±0.5 (*p* = 0.04)*	5.2±0.6	6.0±0.8
*PL54*	123	2.7±1.0	3.3±1.1	3.9±1.2	4.7±1.2

survival from the data in Figure 1 was determined using the one-way ANOVA test. One asterisk (*, significance compared to N2 control RNAi) and two asterisks (**, significance compared to PL51 control RNAi) denote statistical significance at the indicated p-value. The total number of adult nematodes (n) scored for survival in three replicates is indicated. Values represent the day (± standard deviation) at which specified survival for each strain was observed.

We found that methyltransferase-induced increase of survival was greatly limited by the RNAi knockdown of *daf-16*. Although small lifespan decreases were observed in wild-type and *PL54* strains upon *daf-16* RNAi feeding as compared to control RNAi, a much more pronounced reduction in survival was measured in the *PL51* overexpressor strain in those conditions (Fig. 1 and Table 2). In the *PL51* strain, median and maximum lifespan extensions over wild-type values were reduced from 38% and 51% on control RNAi to only 10 and 22% on *daf-16* RNAi. These results show that the lifespan extension observed in PCM-1 overexpressing animals is at least partially dependent on *daf-16* expression levels.

### PCM-1 overexpression enhances resistance to severe thermal stress

**Figure 2:**
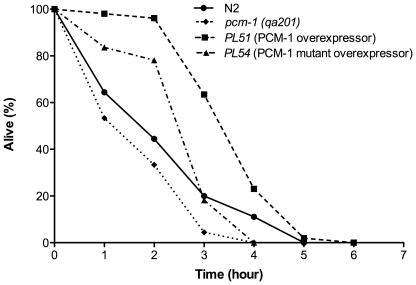
PCM-1 overexpression enhances resistance to severe heat stress (37°C). Survival assays (three trials) were completed for four nematode strains (N2 wild-type, *pcm-1* mutant *(qa201)*, PCM-1 overexpressor strain (*PL51*), mutant PCM-1 overexpressor strain (*PL54*)). Shown are representative survival curves from one trial. L4 larvae were transferred to NGM + OP50 plates and were allowed to grow at 20°C overnight. The next day, animals were transferred to 37°C and survival was scored every two hours for the first four hours and every hour afterwards until all nematodes were dead. Statistical analysis of these data is given in Table 3 for the three replicates of this experiment.

In addition to displaying extended adult lifespans, *daf-2* pathway mutants also show resistance to a variety of stressors, including severe thermal stress [32]. In testing the *PL51* PCM-1 overexpressor strain for resistance to heat stress, we found that day 1 adults displayed increased thermotolerance at 37°C compared to wild-type (N2) animals, most significantly at the 25% survival time point (Fig. 2, Table 3). *PL51* adult animals displayed 25% survival at 4.5±0.5 h compared to 3.2±0.8 h in N2 animals. The enhanced thermotolerance appeared to be at least partially dependent on a functional methyltransferase cofactor-binding domain, as the *PL54* animals did not show significantly increased survival at any noted time point. Furthermore, *pcm-1* mutant animals displayed a significant decrease in thermotolerance when compared to *PL51* animals at all 50%, 25%, and 10% survival time points (Table 3).

**Table 3:** PCM-1 overexpression enhances resistance to severe thermal stress (37°C) in *C. elegans*.

Strain	n	50% Survival (Hours)	25% Survival (Hours)	10% Survival (Hours)	Maximum Lifespan (Hours)
N2	152	2.3±0.9	3.2±0.8	4.2±1.1	5.0±0.8
*pcm-1 (qa201)*	136	1.7±0.6 (*p = *0.04)**	2.8±0.6 (*p* = 0.003)**	3.3±1.0 (*p* = 0.01)**	4.0±1.0
*PL51*	142	3.7±0.6	4.5±0.5 (*p* = 0.04)*	5.2±0.6	6.0±0.8
*PL54*	123	2.7±1.0	3.3±1.1	3.9±1.2	4.7±1.2

Significance of survival from the data in Figure 2 was determined using a one-way ANOVA test. One asterisk (*, significance compared to N2) and two asterisks (**, significance compared to *PL51*) denote statistical significance at the indicated *p-*value. The total number of adult nematodes (n) scored for survival in four replicates is indicated. Values represent the hour (± standard deviation) at which specified survival for each strain was observed.

Preliminary experiments using *daf-16* RNAi indicates that the increased resistance to acute thermal stress of the *PL51* overexpressor strain is, unlike the lifespan extension at 25°C, DAF-16 independent (Figure S2). To investigate this DAF-16 dependency under thermal stress further, we examined the effect of PCM-1 deficiency on the subcellular localization of this transcription factor at 25°C and 37°C. *TJ356* nematodes (which express a GFP-tagged DAF-16 protein) were fed bacteria expressing scrambled control RNAi or *pcm-1* RNAi. Following incubation at 25°C for 48 hours or at 37°C for two hours, the cellular localization of DAF-16 was observed (Figure S3). In our experiments at 25°C, DAF-16::GFP gave a diffuse signal indicating its presence in the cytosol as well as in the nucleus, concurring with results shown recently by Kawli and colleagues (37). No difference in DAF-16 subcellular localization was observed between control RNAi- or *pcm-1* RNAi-fed animals. This is in agreement with the absence of a lifespan phenotype of the *pcm-1* mutant at 25°C. On control RNAi at 37°C, DAF-16 expression was mostly nuclear, confirming previous studies [28] (Figure S3). A similar nuclear localization was observed for DAF-16 on *pcm-1* RNAi after incubation at 37°C (Figure S3). Thus, PCM-1 deficiency does not affect DAF-16 localization under thermal stress. This indicates that the decreased resistance of the *pcm-1* mutant to acute thermal stress that we observe here is not mediated by DAF-16. Further work will be needed to determine whether PCM-1 overexpression, unlike PCM-1 deficiency, affects DAF-16 localization.

### PCM-1 overexpression activates DAF-16-dependent gene expression

Since the results from our lifespan analyses under mild thermal stress showed that the extended lifespan in PCM-1 overexpressing animals is dependent on *daf-16* expression, we next wanted to determine whether PCM-1 overexpression modulates transcription of DAF-16 target genes previously linked to nematode longevity. cDNA was synthesized from total RNA extracted from synchronized day 1 adults (grown at 25°C) and was used to measure expression levels of *pcm-1*, *daf-16*, five genes previously shown to be up-regulated by DAF-16 (*sod-3, hsp-12.6*, *dod-3*, *F21F3.3*, and *mtl-1)* [35], [38], and two genes previously shown to be down-regulated by DAF-16 (*dod-22* and *dod-24)* [35], [38] by real-time qPCR. Transcript levels for these experimental genes were normalized to the transcript levels of two reference genes (*cdc-42* and *ama-1*) in all of the cDNA samples included in the study. *Cdc-42* and *ama-1* were previously shown to be relatively stably expressed in various larval stages and *daf-2* pathway mutants [33] and those genes can thus be considered as reliable reference genes for expression studies in the context of insulin-like signaling in *C. elegans*.

**Figure 3:**
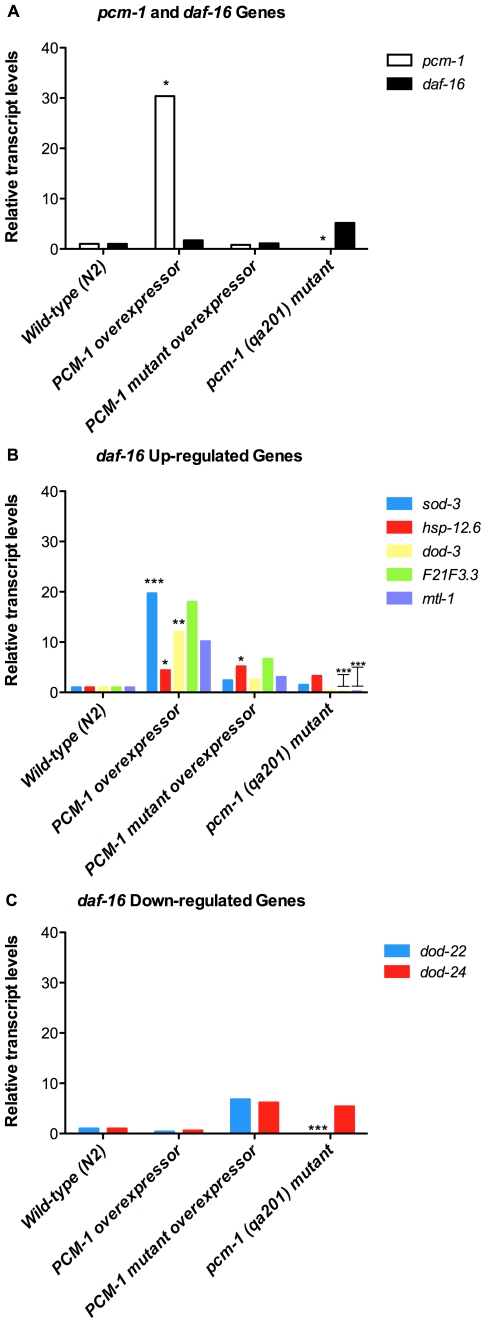
PCM-1 modulates expression levels of DAF-16 target genes at 25°C. Fold changes for the expression of various genes in nematode strains *PL51* (PCM-1 overexpressor), *PL54* (mutant PCM-1 overexpressor), and *pcm-1* (*qa201)* mutant (*pcm-1* null) versus wild-type (N2) are shown. Total RNA was extracted from animals grown at 25°C. Relative transcript levels versus N2 are shown for *pcm-1* and *daf-16* genes (panel A), DAF-16 up-regulated genes (panel B), and DAF-16 down-regulated genes (panel C). *Ama-1* and *cdc-42* were used as endogenous reference genes for normalization of the transcript levels of all of the genes involved in this expression study. Normalized -fold changes and statistical significance were determined using the Qiagen REST 2009 program (*p≤0.05, **p≤0.001, ***p≤0.0001) as shown in Table IV.

As expected, *pcm-1* transcript levels in the overexpressor strain *PL51* were significantly higher than in the wild-type strain (30-fold increase versus wild-type; Fig. 3A). *Pcm-1* transcript levels were reduced by about 500-fold versus wild-type in the *pcm-1* deletion strain *qa201* (Fig. 3A, Table 4). Surprisingly, in the mutant overexpressor strain *PL54*, *pcm-1* transcript levels were not increased above wild-type levels as in the *PL51* strain. However, PCR amplification of exon 3 (i.e. the region which contains the two point mutations that decrease AdoMet binding) from *PL54* cDNA and subsequent sequencing confirmed that this strain expresses a *pcm-1* transcript encoding the expected mutant methyltransferase. The reason for the low expression levels of this mutated transcript as compared to the non-mutated one in the *PL51* strain remains presently unclear. It can be noted here that the plasmids used for creating the transgenic *PL51* and *PL54* strains (in a *pcm-1* mutant background) also contained the *C10F3.4/mcp-1* gene, which overlaps the *pcm-1* gene in the *C. elegans* genome in antiparallel orientation [17], [26]. We found that the *C10F3.4/mcp-1* gene is overexpressed to similar extents in both the *PL51* and *PL54* strains (data not shown). This indicates that the difference in *pcm-1* transcript levels between the two strains results from an effect of the mutations present in the *pcm-1* transgene in the *PL54* strain (but not the *PL51* strain), rather than from a large difference in copy number of transgene DNA in the extrachromosomal array present in the two strains. Despite lower *pcm-1* transcript levels compared to the *PL51* strain, the *PL54* strain thus remains an important control strain to exclude that phenotypes observed in the *PL51* strain are due to *C10F3.4/mcp-1* overexpression. The latter gene encodes for a GDP-d-glucose phosphorylase that has been proposed to function in sanitizing the nucleotide sugar pool [39]. No significant changes in *daf-16* transcript levels were observed in any of the transgenic or mutant strains compared to wild-type levels (Fig. 3A, Table 4).

**Table 4:** PCM-1 modulates expression of DAF-16 target genes under mild thermal stress (25°C).

Strain	Gene	Fold Change Versus N2	Fold Change Versus N2	Fold Change Versus N2	Fold Change Versus N2	Average Fold Change	Standard Deviation	95% Confidence Interval	*P*-value (Versus N2)	*P*-value (Versus *pcm-1 (qa201*))
		**Trial #1**	**Trial #2**	**Trial #3**	**Trial #4**					
***PL51*** ** overexpressor strain**	*pcm-1*	28.0	22.0	41.1		30.4	9.8	9.6 – 98.5	0.03*	
	*daf-16*	0.9	0.5	3.7		1.7	1.8	0.02 – 135.6	0.9	
*Daf-16 up-regulated Genes*	*sod-3*	27.0	14.4	17.7		19.7	6.5	6.5 – 65.3	0.0001***	
	*hsp-12.6*	5.0	2.6	5.7		4.4	1.6	1.7 – 11.2	0.03*	
	*dod-3*	26.0	2.2	3.9	16.0	12.0	11.2	1.1 – 35.9	0.001**	
	*F21F3.3*	55.7	3.2	2.1	11.0	18.0	25.4	0.5 – 69.9	0.06	
	*mtl-1*	24.3	1.9	3.4	11.3	10.2	10.2	0.9 – 28.4	0.07	
*Daf-16 down-regulated Genes*	*dod-22*	0.2	0.7			0.4	0.4			
	*dod-24*	0.4	0.8			0.6	0.3			
***PL54*** ** mutant overexpressor strain**	*pcm-1*	0.9	1.0	0.5		0.8	0.3	0.42 – 1.2	0.5	0.09
	*daf-16*	0.8	0.6	1.8		1.1	0.6	0.02 – 163.3	0.8	0.9
*Daf-16 up-regulated Genes*	*sod-3*	3.3	2.8	1.0		2.4	1.2	0.9 – 4.5	0.1	0.05
	*hsp-12.6*	4.6	9.4	1.5		5.2	4.0	1.6 – 10.8	0.04*	0.3
	*dod-3*	2.5	0.3	1.7	5.7	2.6	2.3	0.3 – 5.9	0.3	0.1
	*F21F3.3*	9.2	1.0	4.4	12.1	6.7	4.9	0.6 – 19.4	0.06	0.004*
	*mtl-1*	2.0	0.3	1.0	9.2	3.1	4.1	0.20 – 8.4	0.6	0.03*
*Daf-16 down-regulated Genes*	*dod-22*	8.3	5.3			6.8	2.1			
	*dod-24*	5.5	6.9			6.2	1.0			
***pcm-1 (qa201)*** ** mutant strain**	*pcm-1*	0.001	0.006	0.000		0.002	0.003	0.000 – 0.006	0.03*	
	*daf-16*	0.04	1	14.6		5.2	8.1	0.04 – 29.7	0.9	
*Daf-16 up-regulated Genes*	*sod-3*	0.6	3.3	0.7		1.5	1.5	0.3 – 3.1	0.9	
	*hsp-12.6*	1.3	7.3	1.2		3.3	3.5	0.8 – 6.9	0.1	
	*dod-3*	0.3	0.2	0.8		0.4	0.3	0.2 – 1.1	0.2	
	*F21F3.3*	0.04	0.1	0.03		0.1	0.0	0.008 – 0.2	0.0001***	
	*mtl-1*	0.2	0.1	0.3		0.2	0.1	0.06 – 0.6	0.0001***	
*Daf-16 down-regulated Genes*	*dod-22*	0.01	0.04	0.04		0.0	0.0	0.006 – 0.2	0.0001***	
	*dod-24*	1.3	11.3	3.7		5.4	5.2	0.9 – 11.1	0.2	

Normalized relative transcript levels for the indicated genes and worm strains compared to wild-type (N2) animals were determined after growth at 25°C. *Ama-1* and *cdc-42* were used as endogenous reference genes for normalization. Fold changes and statistical parameters were calculated using the Qiagen REST 2009 program (*p≤0.05, **p≤0.001, ***p≤0.0001).

The changes in transcript levels measured for DAF-16 target genes correlated with PCM-1 activity levels. Among the “DAF-16 up-regulated” genes, all five genes tested were up-regulated (4- to 20-fold increases vs. wild-type) in PCM-1 overexpressing animals, the increase being statistically significant for *sod-3*, *hsp-12.6*, and *dod-3* (Fig. 3B, Table 4). Much less pronounced increases in transcript level versus wild-type level were observed for these “DAF-16 up-regulated” genes in the mutant overexpressor *PL54* strain, except for the *hsp-12.6* gene, for which similar increases were observed for the *PL51* and *PL54* strains. None of the five “DAF-16 up-regulated” genes was significantly up-regulated versus wild-type in the mutant *pcm-1* strain and two of those genes (*F21F3.3* and *mtl-1*) were actually significantly down-regulated in this strain (Fig. 3B, Table 4). As for the two “DAF-16 down-regulated” genes tested, a down-regulation was also observed in the long-lived PCM-1 overexpressor for both the *dod-22* and *dod-24* genes (about 2-fold decrease versus wild-type for both genes, Fig. 3C, Table 4). On the opposite hand, increases rather than decreases in expression were measured for those genes in the mutant overexpressor *PL54* strain. In the mutant *pcm-1* strain, *dod-22* and *dod-24* expression was down-regulated and up-regulated versus wild-type, respectively (Fig. 3C, Table 4). We should note that the regulation of the mRNA levels of the genes examined here may involve transcription factors in addition to DAF-16; changes in the expression of *pcm-1* may also affect *daf-16-*independent pathways.

### PCM-1 overexpression may not lead to decreased protein damage in *C. elegans*

Accumulation of damaged (isoaspartyl-containing) proteins has been clearly demonstrated over a wide range of molecular weights and in various tissues for isoaspartyl methyltransferase-deficient mice [12]. To better characterize the protein repair function of PCM-1 in *C. elegans*, we quantified the level of substrate proteins for the L-isoaspartyl methyltransferase in extracts prepared from wild-type, *PL51*, and *pcm-1 (qa201)* mutant strains. Therefore, we labeled worm protein extracts (prepared from liquid cultures containing a mixed larval and adult population grown for 5 days at 20°C) with [^3^H]AdoMet and recombinant human isoaspartyl methyltransferase and then separated the polypeptides by SDS polyacrylamide gel electrophoresis at pH 7 for analysis of [^3^H]-methyl ester-containing proteins in individual gel slices as described in the Methods section.

**Figure 4:**
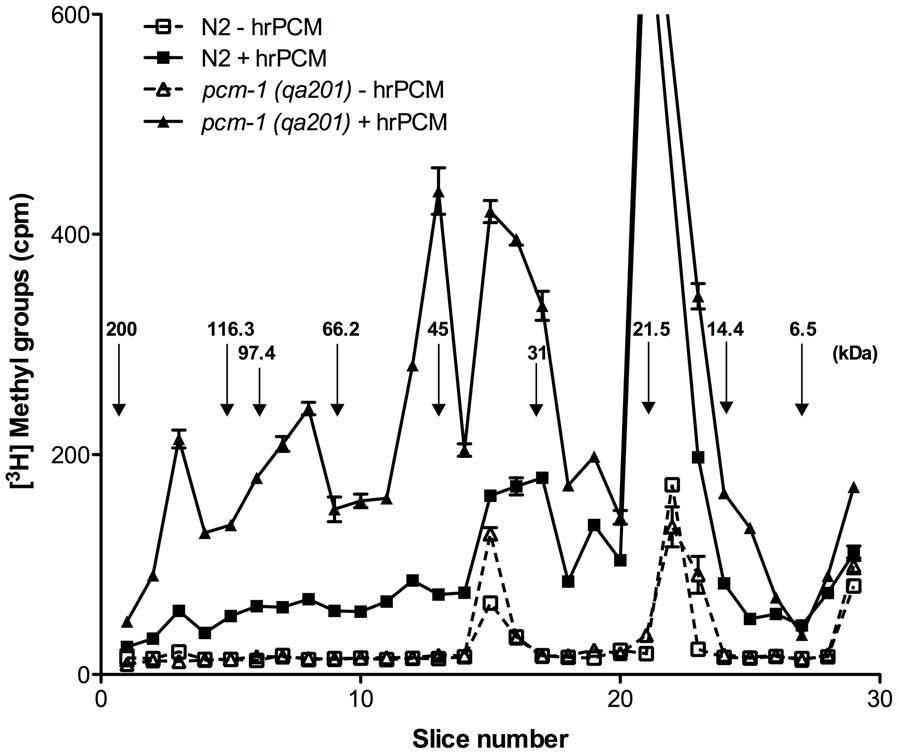
Quantification of methyl-accepting protein substrates in *C. elegans* extracts for the recombinant human L-isoaspartyl methyltransferase at 20°C. Protein extracts from N2 and *pcm-1 (qa201)* nematode strains were prepared from a mixed stage nematode population after liquid culture for 5 days at 20°C and incubated with [^3^H]AdoMet and without or with recombinant human L-isoaspartyl methyltransferase as described in the Methods section. Base-labile methyl esters were quantified in wild-type (N2 - hrPCM, dashed line with open rectangles; N2 + hrPCM, solid line with closed rectangles) and *pcm-1 (qa201*) mutant (*pcm-1 (qa201*) - hrPCM, dashed line with open triangles; *pcm-1 (qa201*) + hrPCM, solid line with closed triangles) strains after polypeptide fractionation by SDS polyacrylamide gel electrophoresis. The migration positions of the molecular weight markers in kDa are indicated by arrows (Biorad SDS-PAGE Standards Catalog #161-0317). Base-labile [^3^H]-methyl ester groups present in peak 22 (not visible in figure) for N2 + hrPCM and *pcm-1 (qa201*) + hrPCM are 7285 cpm and 3683 cpm, respectively. Similar results were obtained in three independent experiments; one representative experiment is shown here and two others in Figure S4.

In three independent experiments, we found significantly higher levels of base-labile [^3^H]-methyl ester groups in *pcm-1 (qa201)* mutant extracts compared to wild-type extracts prepared from nematodes grown at 20°C (Figs. 4 and S4) for proteins of molecular weights ranging from about 25 to 200 kDa. However, for reasons that presently remain unclear, in two other experiments of the same type we did not observe as significant of an increase in protein damage in the *pcm-1* mutant (Figure S5, top panel). For the two assays performed on extracts derived from nematodes grown at 25°C, we did not observe significantly higher isoaspartyl levels in *pcm-1* mutant protein extracts than in wild-type extracts (Figure S5, bottom panel). Further work is necessary to understand the reason underlying the variability of these results, but they indicate that *pcm-1* mutant animals display a tendency to accumulate higher protein isoaspartyl levels. As *pcm-1* mutant *C. elegans* do not present any lifespan phenotype at either 20°C or 25°C, these results indicate that isoaspartyl damage might not affect lifespan in nematodes. In all of our labeling experiments we generally observed a major peak of radioactive methyl group incorporation at 21 kDa, but the level of this incorporation seemed to be independent of PCM-1 activity.

We also measured isoaspartyl levels in protein extracts derived from PCM-1 overexpressing nematodes at both 20°C or 25. Our results showed no decrease in base-labile [^3^H]-methyl ester groups below basal wild-type levels in the PCM-1 overexpressing (*PL51*) extracts derived from nematodes grown at either 20°C or 25°C. On the contrary, equal or variably increased amounts of damaged proteins in *PL51* animals versus wild-type animals were observed (data not shown). This indicates that a several-fold increase in isoaspartyl methyltransferase activity over wild-type activity levels does not decrease isoaspartyl levels below those accumulating in wild-type animals and suggests that the lifespan extension displayed by PCM-1 overexpressing animals does not result from a decrease in overall protein damage (in agreement with the above conclusion that increased protein damage does not affect lifespan).

## Discussion

We show here that PCM-1 overexpression extends nematode lifespan under mild thermal stress (25°C) in a DAF-16-dependent manner. This finding is in agreement with previous results indicating that PCM-1 activity in adult nematodes might be dispensable under control conditions, but becomes critically important under various stress conditions or in specific larval stages [17], [25], [26]. Similarly, in preliminary experiments carried out in preparation of this study, no lifespan extension over wild-type was observed in PCM-1 overexpressing nematodes when grown at 20°C.

**Figure 5:**
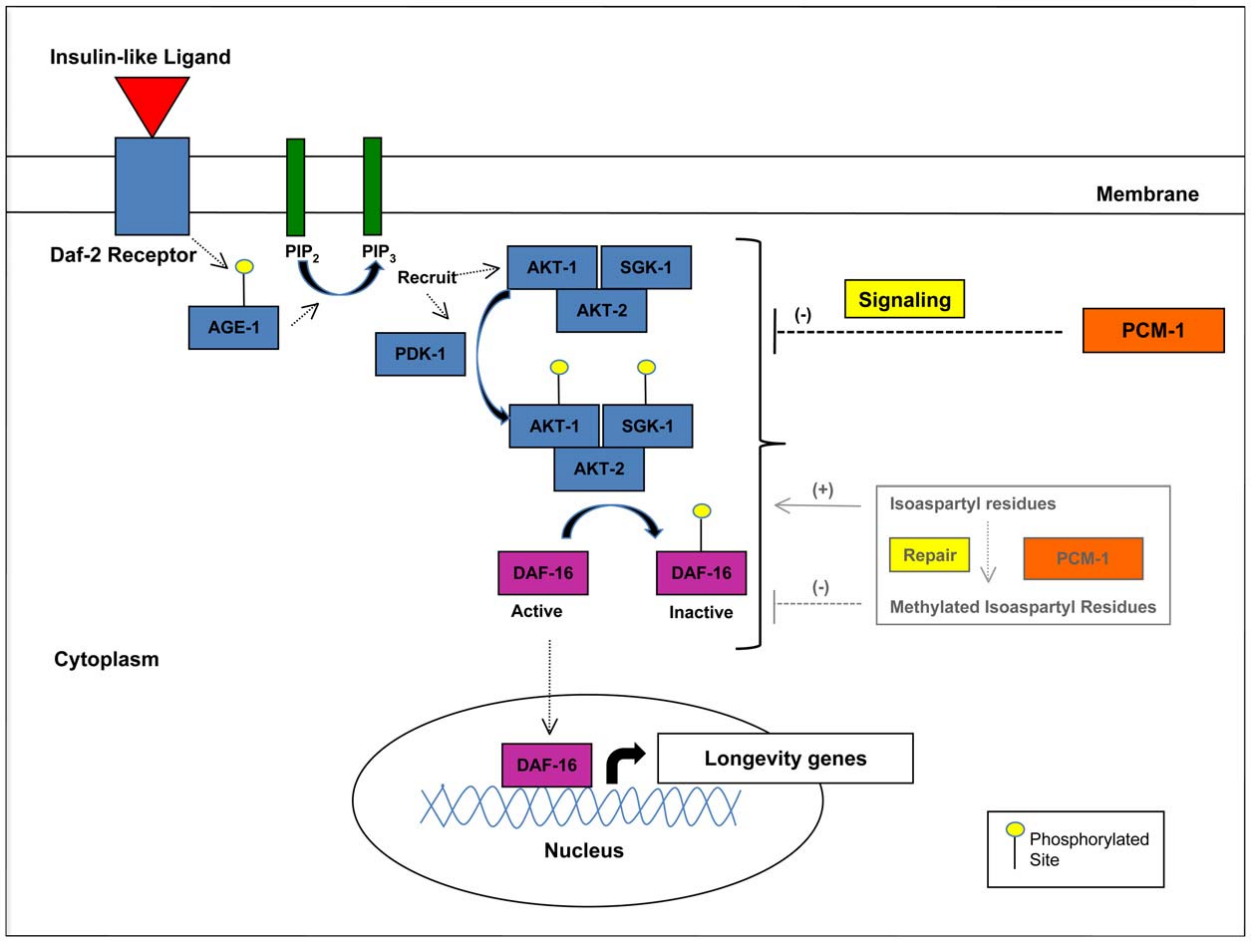
The protein L-isoaspartyl *O*-methyltransferase (PCM-1) opposes insulin-like signaling to extend *C. elegans* lifespan. Upon binding of the insulin-like ligand to the DAF-2 receptor, the AGE-1 kinase is activated and phosphorylates membrane-bound phosphatidylinositol-4,5-bisphosphate (PIP_2_ ), leading to the formation of phosphatidylinositiol-3,4,5-trisphosphate (PIP_3_) and recruitment of kinases PDK-1 and AKT-1/AKT-2/SGK-1 complex to the membrane. PDK-1 subsequently phosphorylates and activates the AKT/SGK complex, leading to further phosphorylation and inactivation of the FoxO transcription factor DAF-16. In the absence of phosphorylation, DAF-16 enters the nucleus to induce transcription of genes implicated in stress resistance and longevity. Our results indicate that overexpression of the protein repair enzyme PCM-1 leads to down-regulation of the insulin-like signaling pathway and subsequent activation of DAF-16, leading to increased transcription of longevity genes. Our results also show that, although absence of PCM-1 activity leads to increased accumulation of protein damage, its overexpression might not decrease the level of isoaspartyl-containing proteins beyond wild-type levels. This suggests that PCM-1 might control lifespan in *C. elegans* through a regulatory signaling function rather than through its role in bulk protein repair.

By employing *daf-16* RNAi, we were able to show that a reduction in *daf-16* expression partially reduced the lifespan extension displayed by the *PL51* overexpressor strain at 25°C. *Daf-16* RNAi also decreased lifespan in the mutant overexpressor animals, but this reduction was much less pronounced and mimicked the one observed in wild-type animals. A small negative effect of *daf-16* RNAi on lifespan in N2 animals has been reported previously [40], [41]. Our results thus indicate that the PCM-1 isoaspartyl methyltransferase extends lifespan at 25°C at least partially by directly or indirectly downregulating the IIS pathway and thereby promoting nuclear translocation of DAF-16 with concomitant up-regulation of target genes promoting survival under mild thermal stress (Figure 5).

The partial nature of the reduction of the PCM-1-dependent lifespan extension by *daf-16* RNAi suggests that PCM-1 might also act independently of *daf-16* to increase lifespan. Possible targets include the transcription factor SKN-1 [42], [43]. SKN-1 functions in parallel to DAF-16 to promote longevity and is also a direct target of IIS; in response to oxidative or xenobiotic stress, SKN-1 induces a set of stress response genes and reduced IIS leads to nuclear accumulation and transcriptional activation of SKN-1 in the intestine [42]. Alternatively, complete suppression of the lifespan extension may require *daf-16* down-regulation also in the neurons. It has been shown that RNAi does not penetrate neurons effectively in *C. elegans* animals [44], [45]; we have confirmed this result here by finding that *daf-16* RNAi treatment does not affect DAF-16 expression in neurons. Although DAF-16 activation occurs in both neuronal and intestinal cells, it has been shown that DAF-16 activity specifically in the intestine increases the lifespan of *daf-2*;*daf-16* double mutants substantially [46], [47]. However, it has also been shown that lifespan extension in *age-1* mutants requires both neuronal and intestinal DAF-16 activity [47], [48]. Thus, it is possible that the small lifespan extension we observe with PCM-1 overexpression in the *daf-16* RNAi treated adult nematodes is a result of DAF-16 activation in neurons. It is also known that PCM-1 is expressed primarily in the neurons, body wall, and reproductive tissues in *C. elegans* [25]. We thus speculate that a complete suppression of the *daf-16*-dependent lifespan extension triggered by PCM-1 overexpression might only be achieved in neuronal RNAi-sensitive worm strains.

We also observed an increase in median survival under severe thermal stress (37°C) in the overexpressor strain that is dependent on a functional L-isoaspartyl methyltransferase. Preliminary examination of the enhanced thermal stress resistance in the *PL51* strain indicates that the increased thermotolerance observed is *daf-16*-independent (Figure S2). In agreement with this, our preliminary microscopy results show that *pcm-1* RNAi does not suppress nuclear localization of DAF-16 during heat shock at 37°C (Figure S3). Thus, increased survival of the *PL51* nematodes at 37°C might involve a DAF-16-independent mechanism.

Real-time qPCR analyses showed a PCM-1-dependent induction of the DAF-16 target genes *sod-3*, *F21F3.*3, *mtl-1,* and *dod-3* at 25°C. The 20-fold induction of *sod-3* transcription and the approximately 10-fold to 20-fold induction of the *dod-3*, *F21F3.3*, and *mtl-1* genes over wild-type levels in the PCM-1 overexpressor strain at 25°C further solidifies our hypothesis of PCM-1 downregulating IIS signaling to extend nematode longevity under mild thermal stress. We note that *mtl-1* expression can also be regulated by the ELT-2 transcription factor [49]. At this point, we cannot rule out effects of PCM-1 on DAF-16-independent pathways. Although the bulk of our evidence points to the linkage of PCM-1 activity to the activity of DAF-16, it is possible that PCM-1 can affect DAF-16 target genes through other transcription factors.

Surprisingly, the mutant overexpressor strain *PL54* did not overexpress *pcm-1* to the same extent as the wild-type overexpressor strain *PL51*. We confirmed the presence of the expected point mutations in the *pcm-1* transcript in the *PL54* strain, and consequently believe that the mutant overexpressor strain expresses a non-functional PCM-1 protein at lower levels, as our activity assays confirm that there is no PCM-1 activity in this strain. It was interesting to observe that a few of the “DAF-16 up-regulated” genes were also significantly up-regulated compared to wild-type animals in the *PL54* mutant overexpressor strain. Except for *hsp-12.6*, for which an approximate 5-fold induction was observed in both *PL54* and *PL51* strains, the levels of induction of the other DAF-16 up-regulated genes were, however, much lower in the *PL54* mutant overexpressor strain than those reached in the *PL51* wild-type overexpressor strain. These effects may be attributed to the regulation of *hsp-12.6* by the HSF-1 transcription factor (50) or by effects of the *C10F3.4/mcp-1* gene which overlaps the *pcm-1* gene in antiparallel orientation and which is overexpressed to the same extent in both the *PL51* and *PL54* transgenic strains.

Concerning the “DAF-16 down-regulated” genes analyzed in our expression study (*dod-22* and *dod-24*), the results obtained in the PCM-1 transgenic strains (about 2-fold down-regulation in the *PL51* strain as opposed to an about 6- to 7-fold up-regulation in the *PL54* strain) also support our hypothesis of a PCM-1 mediated downregulation of the IIS. However, whereas the *dod-24* gene was up-regulated to a similar extent in the *PL54* mutant overexpressor strain as in the *pcm-1* deletion strain, a more puzzling result was the very low expression measured for *dod-22* in the latter strain. It might be noted here that the status of the *dod-22* and *dod-24* genes as “downstream of DAF-16” targets is presently controversial. Initial work by Murphy and colleagues [38] identified *dod-22* and *dod-24* as genes acting downstream of DAF-16 to regulate longevity in *C. elegans*. These genes were also shown to be down-regulated in long-lived TGF-β mutant adult animals and this regulation was dependent on DAF-16 [51]. Additionally, the *dod-22* and *dod-24* genes were used as control down-regulated DAF-16 target genes in a recent study on HCF-1, a negative regulator of DAF-16 [35]. Other recent studies [52], [53] suggest, however, that regulation of *dod-22* and *dod-24* expression is largely independent of DAF-16. *Dod-24* expression was shown to be negatively regulated by the nuclear hormone receptor DAF-12 via signaling from the somatic reproductive tissues and independently of DAF-16 [48] and *dod-22* expression has been shown to be regulated by the p38/MAPK pathway independently of DAF-16 [53]. Both studies show only a partial/restrictive dependence on DAF-16, explaining their presence in the original screen [38]. Given those recent observations on the regulation of *dod-22* and *dod-24* expression, the effects of PCM-1 on the expression levels of those genes might not, or only be partially mediated by DAF-16. Further work is needed to clarify this point.

To determine whether an increase in PCM-1 activity leads to increased protein repair in *C. elegans*, we measured protein isoaspartyl levels in wild-type, *pcm-1* mutant, and *PL51* extracts by using human recombinant isoaspartyl methyltransferase. Our results show an increased isoaspartyl content in *pcm-1* mutant extracts compared to wild-type extracts prepared from nematodes grown in liquid culture, suggesting a defect in the repair of isoaspartyl residues in the absence of methyltransferase. In the PCM-1 overexpressing (*PL51*) extracts, there was no apparent decrease in isoaspartyl damage compared to wild-type levels. However, *PL51* is a non-integrated transgenic strain, with an observed transmission of about 30%. Thus, we estimate that the liquid cultures from which we prepared our extracts contained about 30% PCM-1 overexpressing transgenic animals and 70% non-transgenic *pcm-1* mutant animals. Hence, if the transgenic PCM-1 overexpressing animals had decreased levels of isoaspartyl damage, this change may have been masked by the increased levels of damage in the 70% non-transgenic *pcm-1* mutant nematodes.

Taken together, these results suggest that increased accumulation of protein damage (as observed in mutant *pcm-1* animals) does not affect lifespan (no difference in lifespan between wild-type and *pcm-1* mutant animals) and, conversely, that PCM-1 overexpression might not extend lifespan by decreasing the overall content of damaged proteins in the cell. Rather, PCM-1 may act as a signaling protein to directly or indirectly downregulate the IIS pathway to turn on expression of stress resistance genes via activation of DAF-16 (Figure 5). One possible mechanism could be a posttranslational regulatory carboxyl methylation of an intermediate of the IIS or of a protein phosphatase by PCM-1. The future identification of such potential regulatory targets of PCM-1 will help elucidate the mechanism of the observed interaction between the isoaspartyl methyltransferase, the IIS, and lifespan control in nematodes as well as in higher organisms.

## Supporting Information

Figure S1 ***Pcm-1*** **RNAi reduces the lifespan extension of PCM-1 overexpressing adult nematodes at 25°C.** Lifespan analyses (one trial) were completed at 25°C (with FUDR) for two nematode strains (N2 wild-type (top panel) and wild-type PCM-1 overexpressor strain (*PL51*) (bottom panel)) grown in three conditions: control RNAi (solid lines) and *pcm-1* RNAi (clones #2 and #3, dotted lines). L4 larvae were transferred (Day 0) to NGM plates streaked with RNAi bacteria and survival was scored every other day until all nematodes were dead. Wild-type (N2) animals scored for survival were as follows: control (n = 74) and *pcm-1* RNAi conditions (n = 77 for *pcm-1* RNAi clone #2; n = 76 for *pcm-1* RNAi clone #3). *PL51* animals scored for survival were as follows: control (n = 55) and *pcm-1* RNAi conditions (n = 70 for *pcm-1* RNAi clone #2; n = 70 for *pcm-1* RNAi clone #3).
(TIF)

Figure S2 ***Daf-16*** **RNAi does not significantly affect resistance to severe heat stress (37°C) in PCM-1-overexpressing animals.** Survival assays (one trial) were completed for two nematode strains (N2 wild-type and PCM-1 overexpressor strain (*PL51*)) in control RNAi and *daf-16* RNAi conditions. L4 larvae were transferred to NGM + RNAi plates and were allowed to grow at 20°C overnight. The next day, animals were transferred to 37°C and survival was scored every two hours for the first four hours and every hour afterwards until all nematodes were dead. Wild-type (N2) animals scored for survival were as follows: control (n = 27) and *daf-16* RNAi conditions (n = 26). *PL51* animals scored for survival were as follows: control (n = 15) and *daf-16* RNAi conditions (n = 20).
(TIF)

Figure S3 **Monitoring DAF-16 localization with** ***pcm-1*** **RNAi under mild (25°C) and severe (37°C) thermal stress.** *TJ356* nematode animals were fed bacteria expressing either control (scramble) or *pcm-1* RNAi as described in the Methods sections. DAF-16 localization was monitored using Nomarski differential interference contrast microscopy at 200-fold magnification. The top three panels show mostly diffuse, unlocalized DAF-16 expression in animals fed control RNAi (n = 22), *pcm-1* RNAi clone #2 (n = 25), and *pcm-1* RNAi clone #3 (n = 19) at 25°C. The bottom three panels show nuclear DAF-16 expression in animals fed control RNAi (n = 8), *pcm-1* RNAi clone #2 (n = 12), and *pcm-1* RNAi clone #3 (n = 13) following incubation at 37°C.
(PDF)

Figure S4 **Quantification of protein isoaspartyl levels in additional** ***C. elegans*** **extracts at 20°C**. Protein extracts were prepared from liquid nematode cultures grown at 20°C and analyzed for isoaspartyl content as described in Figure 4. Data from two additional biological replicate lysates are shown in panel A (replicate 1) and panel B (replicate 2). For panel A, base-labile [^3^H]-methyl ester groups present in peak 22 (not visible in figure) for N2+ hrPCM and *pcm-1 (qa201*) + hrPCM are 14,514 cpm and 12,862 cpm, respectively. For panel C, base-labile [^3^H]-methyl ester groups present in peak 26 (not visible in figure) for N2+ hrPCM and *pcm-1 (qa201*) + hrPCM are 247 cpm and 380 cpm, respectively. A less active human recombinant PCM was used in replicate 2 compared to the hrPCM used in the experiments shown in Figure 4 and in panel A of this Figure, but the same increase in base-labile [^3^H]-methyl ester groups in labeled *pcm-1 (qa201)* mutant extracts was observed.
(TIF)

Figure S5 **Quantification of protein isoaspartyl levels in additional** ***C. elegans*** **extracts prepared from nematodes grown at 20°C (top panel) and 25°C (bottom panel)**. Protein extracts were prepared from liquid nematode cultures and analyzed for isoaspartyl content as described in Figure 4. The averages of data from two additional biological replicate lysates (prepared from nematodes grown at 20°C) are shown in the top panel. Base-labile [^3^H]-methyl ester groups present in peak 23 (not visible in figure) for N2+ hrPCM and *pcm-1 (qa201*) + hrPCM are 7,851 cpm and 19,384 cpm, respectively. In the bottom panel, data from two biological replicate lysates (prepared from nematodes grown at 25°C) for both strains are shown. Base-labile [^3^H]-methyl ester groups present in peak 24 (not visible in figure) for N2+ hrPCM and *pcm-1 (qa201*)+ hrPCM are 4,858 cpm and 8,017 cpm, respectively.
(TIF)
