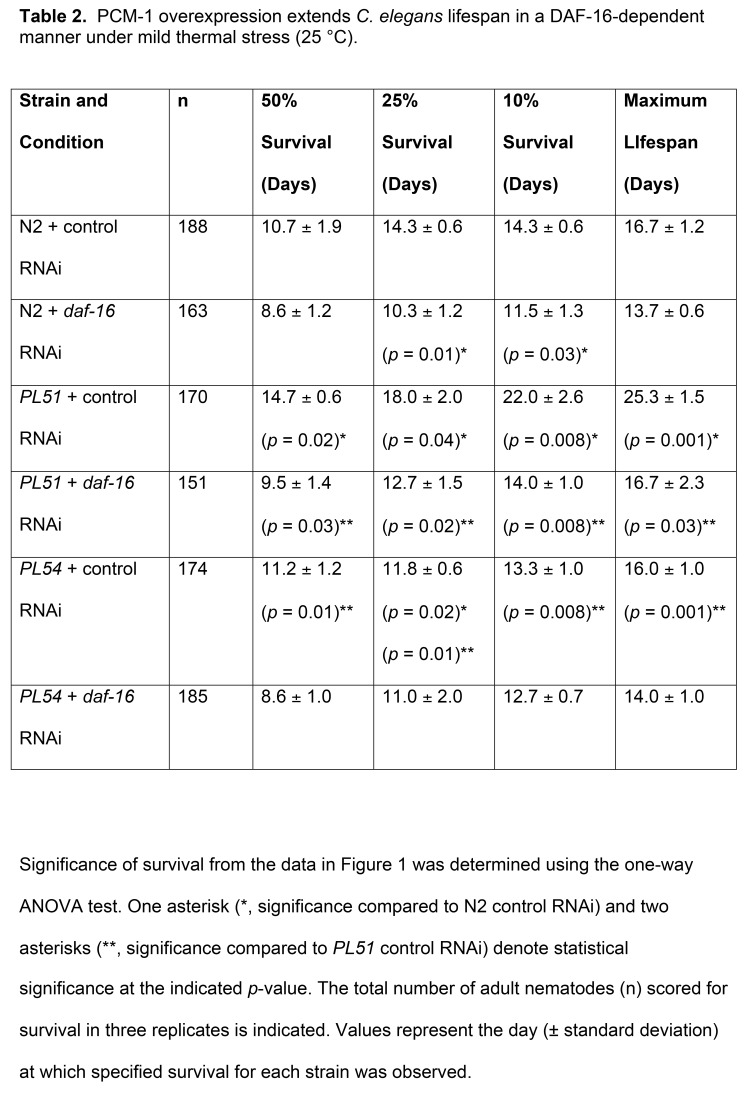# Correction: The Interplay between Protein L-Isoaspartyl Methyltransferase Activity and Insulin-Like Signaling to Extend Lifespan in *Caenorhabditis elegans*

**DOI:** 10.1371/annotation/d6ad8c55-fd3a-4f6c-a2ba-849e4a6500ca

**Published:** 2013-05-03

**Authors:** Shilpi Khare, Carole L. Linster, Steven G. Clarke

Table 2 was replaced by a duplicated version of Table 3. The correct version of Table 3 is available here: 

**Figure:**