# Supplementary figures and images for: The Interplay between Protein L-Isoaspartyl Methyltransferase Activity and Insulin-Like Signaling to Extend Lifespan in Caenorhabditis elegans
Source: PLoS One. 2011 Jun 13;6(6):e20850. doi: 10.1371/journal.pone.0020850 (PMC3113807; doi:10.1371/journal.pone.0020850)

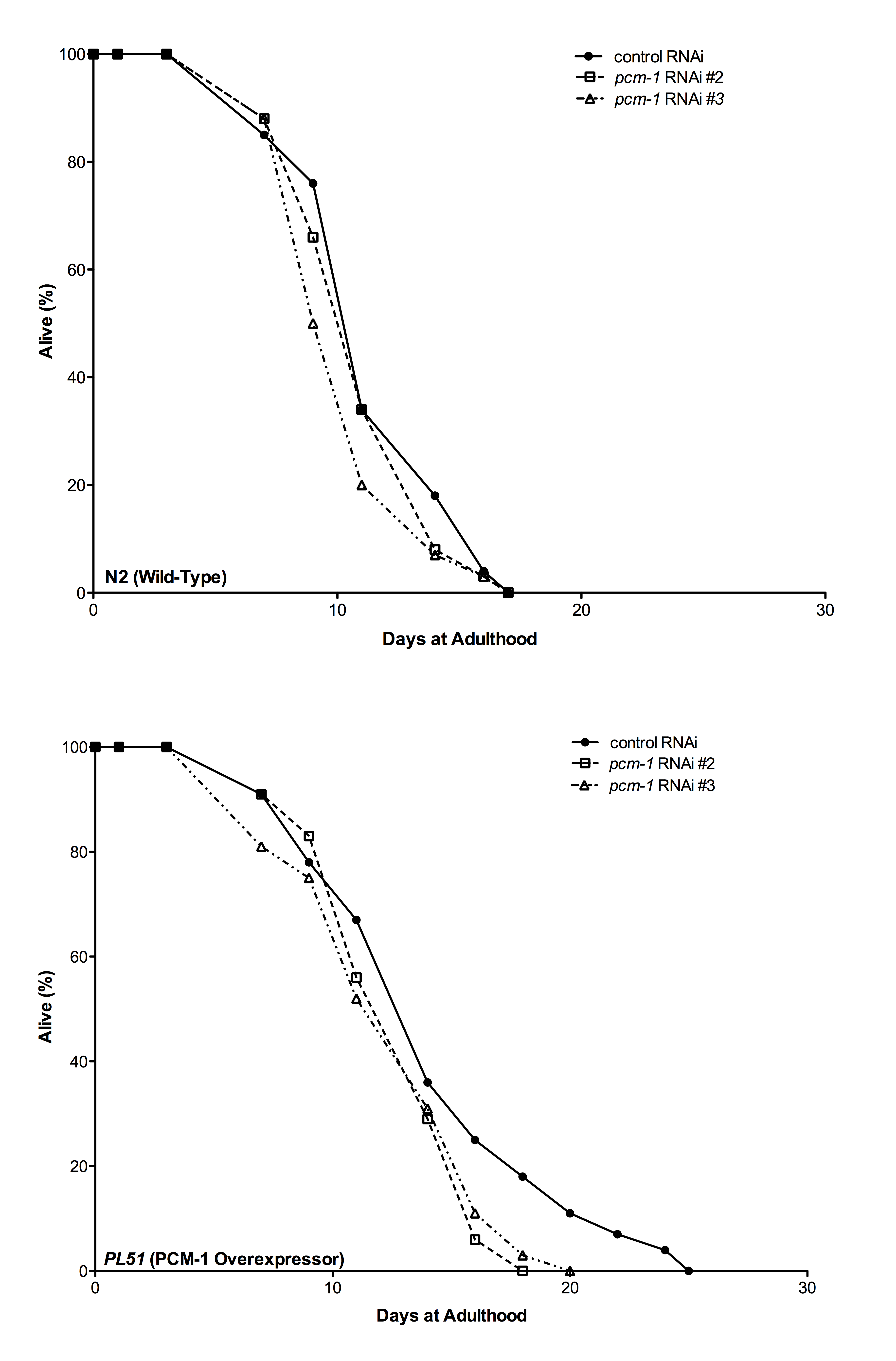

Supplement: Figure S1 — Pcm-1 RNAi reduces the lifespan extension of PCM-1 overexpressing adult nematodes at 25°C. Lifespan analyses (one trial) were completed at 25°C (with FUDR) for two nematode strains (N2 wild-type (top panel) and wild-type PCM-1 overexpressor strain (PL51) (bottom panel)) grown in three conditions: control RNAi (solid lines) and pcm-1 RNAi (clones #2 and #3, dotted lines). L4 larvae were transferred (Day 0) to NGM plates streaked with RNAi bacteria and survival was scored every other day until all nematodes were dead. Wild-type (N2) animals scored for survival were as follows: control (n = 74) and pcm-1 RNAi conditions (n = 77 for pcm-1 RNAi clone #2; n = 76 for pcm-1 RNAi clone #3). PL51 animals scored for survival were as follows: control (n = 55) and pcm-1 RNAi conditions (n = 70 for pcm-1 RNAi clone #2; n = 70 for pcm-1 RNAi clone #3). (TIF) [file pone.0020850.s001.tif]

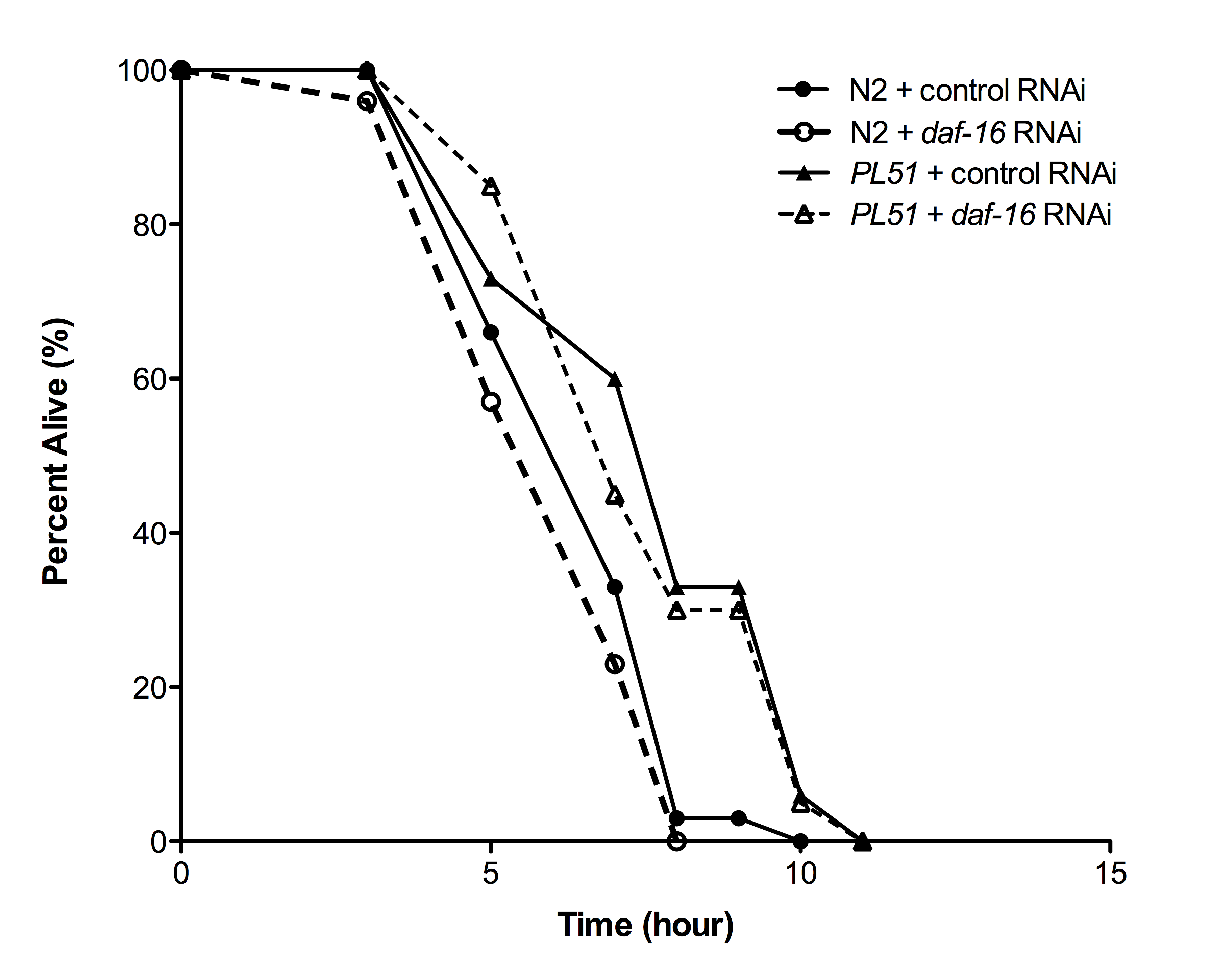

Supplement: Figure S2 — Daf-16 RNAi does not significantly affect resistance to severe heat stress (37°C) in PCM-1-overexpressing animals. Survival assays (one trial) were completed for two nematode strains (N2 wild-type and PCM-1 overexpressor strain (PL51)) in control RNAi and daf-16 RNAi conditions. L4 larvae were transferred to NGM + RNAi plates and were allowed to grow at 20°C overnight. The next day, animals were transferred to 37°C and survival was scored every two hours for the first four hours and every hour afterwards until all nematodes were dead. Wild-type (N2) animals scored for survival were as follows: control (n = 27) and daf-16 RNAi conditions (n = 26). PL51 animals scored for survival were as follows: control (n = 15) and daf-16 RNAi conditions (n = 20). (TIF) [file pone.0020850.s002.tif]

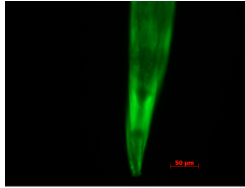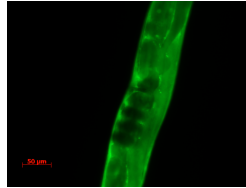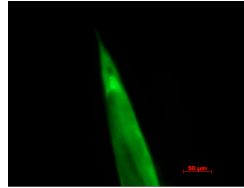

**control RNAi (25 °C)**

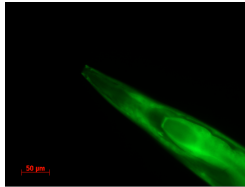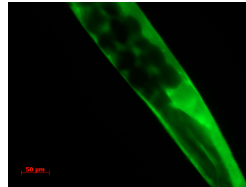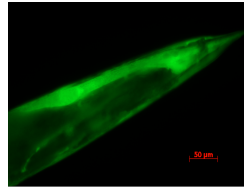

***pcm-1* RNAi (#2) (25 °C)**

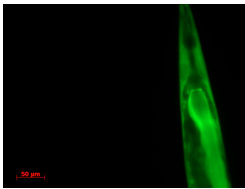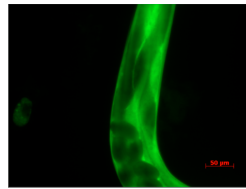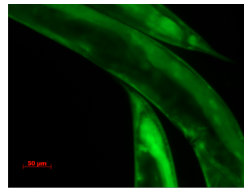

***pcm-1* RNAi (#3) (25 °C)**

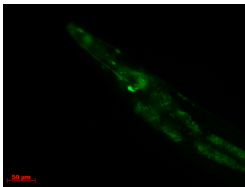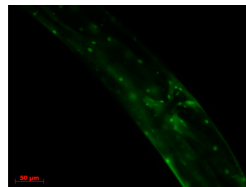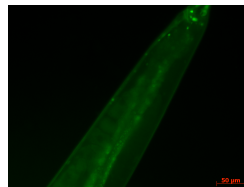

**control RNAi (37 °C)**

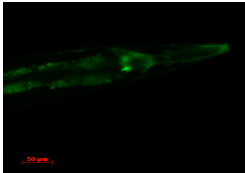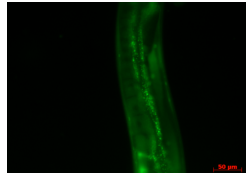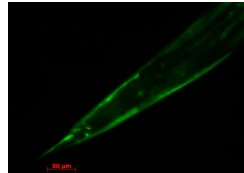

***pcm-1* RNAi (#2) (37 °C)**

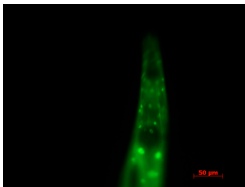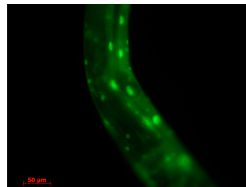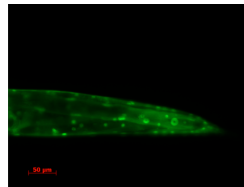

***pcm-1* RNAi (#3) (37 °C)**

Supplement: Figure S3 — Monitoring DAF-16 localization with pcm-1 RNAi under mild (25°C) and severe (37°C) thermal stress. TJ356 nematode animals were fed bacteria expressing either control (scramble) or pcm-1 RNAi as described in the Methods sections. DAF-16 localization was monitored using Nomarski differential interference contrast microscopy at 200-fold magnification. The top three panels show mostly diffuse, unlocalized DAF-16 expression in animals fed control RNAi (n = 22), pcm-1 RNAi clone #2 (n = 25), and pcm-1 RNAi clone #3 (n = 19) at 25°C. The bottom three panels show nuclear DAF-16 expression in animals fed control RNAi (n = 8), pcm-1 RNAi clone #2 (n = 12), and pcm-1 RNAi clone #3 (n = 13) following incubation at 37°C. (PDF) [file pone.0020850.s003.pdf]

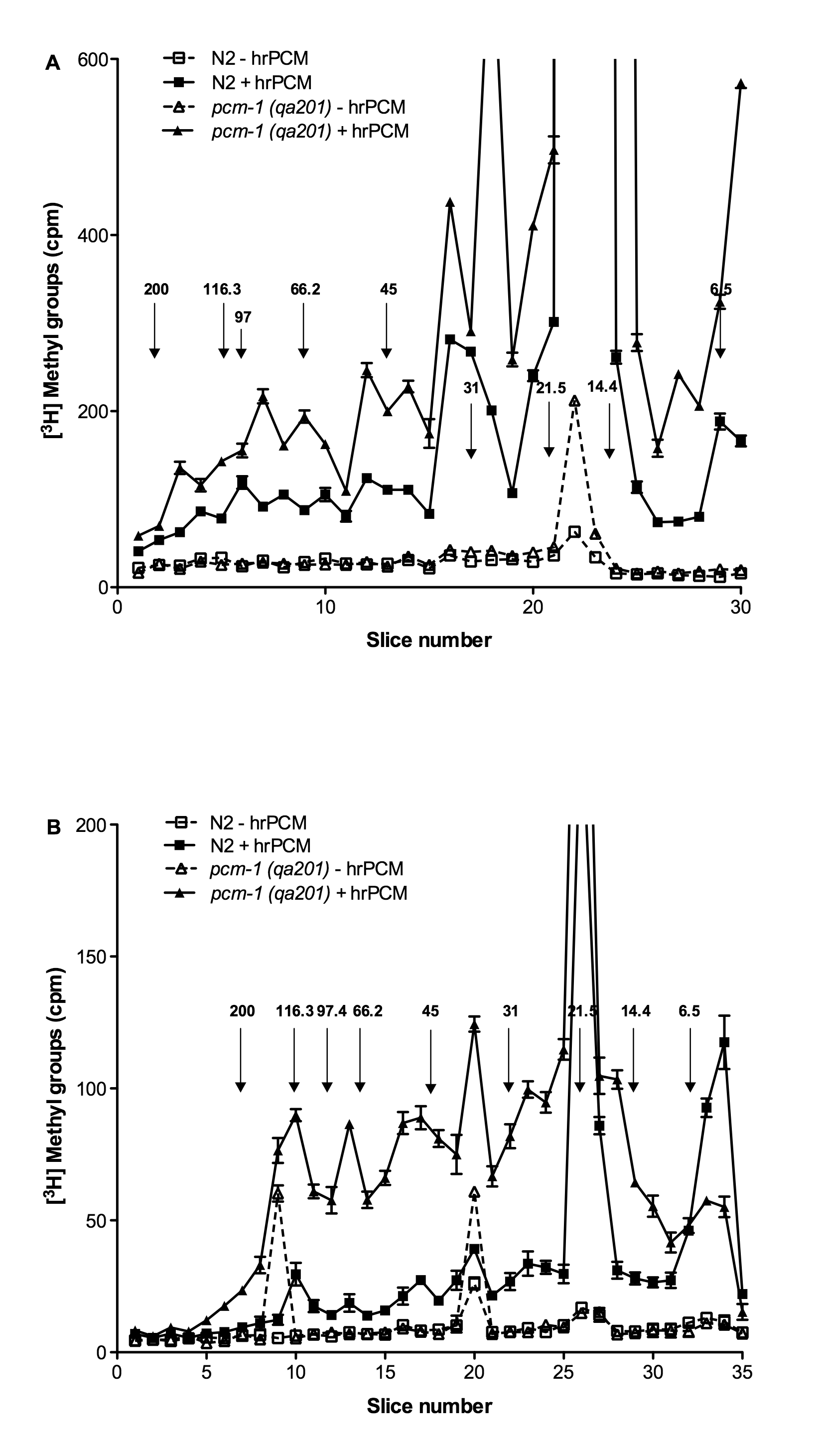

Supplement: Figure S4 — Quantification of protein isoaspartyl levels in additional C. elegans extracts at 20°C. Protein extracts were prepared from liquid nematode cultures grown at 20°C and analyzed for isoaspartyl content as described in Figure 4. Data from two additional biological replicate lysates are shown in panel A (replicate 1) and panel B (replicate 2). For panel A, base-labile [3H]-methyl ester groups present in peak 22 (not visible in figure) for N2+ hrPCM and pcm-1 (qa201) + hrPCM are 14,514 cpm and 12,862 cpm, respectively. For panel C, base-labile [3H]-methyl ester groups present in peak 26 (not visible in figure) for N2+ hrPCM and pcm-1 (qa201) + hrPCM are 247 cpm and 380 cpm, respectively. A less active human recombinant PCM was used in replicate 2 compared to the hrPCM used in the experiments shown in Figure 4 and in panel A of this Figure, but the same increase in base-labile [3H]-methyl ester groups in labeled pcm-1 (qa201) mutant extracts was observed. (TIF) [file pone.0020850.s004.tif]

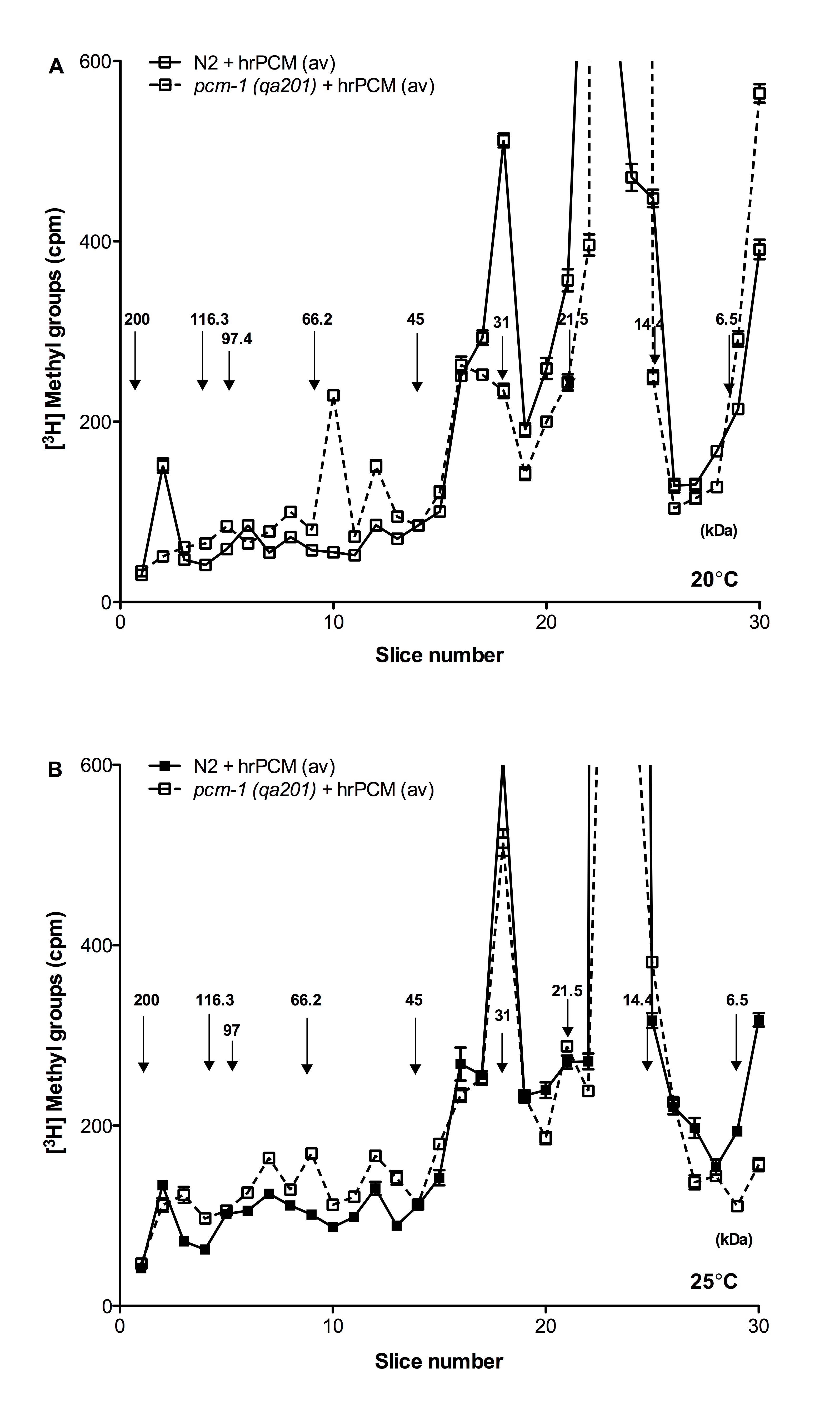

Supplement: Figure S5 — Quantification of protein isoaspartyl levels in additional C. elegans extracts prepared from nematodes grown at 20°C (top panel) and 25°C (bottom panel). Protein extracts were prepared from liquid nematode cultures and analyzed for isoaspartyl content as described in Figure 4. The averages of data from two additional biological replicate lysates (prepared from nematodes grown at 20°C) are shown in the top panel. Base-labile [3H]-methyl ester groups present in peak 23 (not visible in figure) for N2+ hrPCM and pcm-1 (qa201) + hrPCM are 7,851 cpm and 19,384 cpm, respectively. In the bottom panel, data from two biological replicate lysates (prepared from nematodes grown at 25°C) for both strains are shown. Base-labile [3H]-methyl ester groups present in peak 24 (not visible in figure) for N2+ hrPCM and pcm-1 (qa201)+ hrPCM are 4,858 cpm and 8,017 cpm, respectively. (TIF) [file pone.0020850.s005.tif]
